# Potential barriers to the daily use of hearing aids in children

**DOI:** 10.15537/smj.2023.44.4.20220766

**Published:** 2023-04

**Authors:** Medhat F. Yousef, Ziyad I. Dhayan, Tahera Islam, Fahad Z. Alotabi, Eman A Hajr

**Affiliations:** *From the College of Medicine (Yousef), King Abdullah Ear Specialist Center, King Saud University; from King Saud University (Dhayan); from the College of Medicine and Research Center (Islam), King Saud University; from the Department of Otolaryngology (Alotabi, Hajr), Imam Mohammad Ibn Saud Islamic University, Riyadh, Kingdom of Saudi Arabia; from the Audiology Unit, ENT Department (Yousef), Faculty of Medicine, Menoufia University, Shibin El Kom, Menoufia, Egypt.*

**Keywords:** hearing aid, children, hearing loss

## Abstract

**Objectives:**

To identify factors affecting hearing aid usage in children.

**Methods:**

This retrospective study examined 59 hearing-impaired children fitted with hearing aids for at least 6 months. Patients with moderate to profound sensorineural hearing loss with complete data-logging information stored in the hearing aid programming file from January 2020 until June 2021 were included. Children with concomitant disabilities were excluded. Data for audiological assessments included hearing assessment, aided hearing thresholds, and aided speech tests.

**Results:**

The children’s age ranged from 6 months to 6 years. Average daily hearing aid usage was 5.5 hour (h) after 3 months, 7 h after 6 months; and 8.7± 4.7 h as reported by parents. Patient age was positively correlated with data logging at 3 months (r=0.414, *p*=0.01) and 6 months (r=0.406, *p*=0.01).

**Conclusion:**

We found that children’s age, gender, severity of hearing loss, residential location, and parents’ educational level had a significant effect on daily hearing aid usage. Whereas, family size and a family history of hearing loss or use of amplification devices had no discernible influence.

**A**ccording to the World Health Organization, 466 million individuals, comprising 432 million adults and 34 million children, worldwide experience disabling hearing loss. This number is expected to increase to 700 million by 2050.^1^ Although most people with hearing loss can benefit from the use of hearing aids (HAs), there is considerable variation in the improvement of hearing function among individual users.^2^ Additionally, HAs are linked to several issues that may contribute to their non-use, including acoustic feedback, discomfort, inadequate amplification, maintenance requirements, and perceived social stigma.^2^

Early detection and intervention of hearing loss in newborns, particularly in the first 3 months of life, has a tremendous impact on language development and communicative ability.^3^ However, Zhang et al^4^ reported that early hearing screening and HA intervention have certain limitations. They found that insurance status, race, and the native language of participants with hearing loss affect early identification and intervention,^4^ whereas geographical location and medical status did not influence the effectiveness of early intervention. Moreover, the length of daily HA usage, maternal educational status, and children’s cognitive level were reported to affect the efficacy of these therapies.^5,6^ Close monitoring of hearing aid performance and variables influencing their usage in children is an important aspect in assessing eligibility and timing of alternative interventions such as cochlear implantation.^5^

Walker et al^6^ assessed the linguistic development of pediatric HA users aged 5-7 years, who were classified into 3 subgroups according to the duration of HA use. They found that those with prolonged HA use showed considerable improvement in vocabulary and grammatical components of language. Tomblin et al^7^ carried out a longitudinal study of children with mild to severe hearing loss and similarly reported the substantial effect of the duration of HA use on language acquisition and development. In particular, children who used HAs for ≥10 h daily had better language abilities than those who used HAs for fewer hours.^8^

Hearing aids usage in young children is challenging for both parents and audiologists. Various factors may influence children’s sustained use of HAs. Walker et al^9^ reported that older children used the device more consistently than younger children. Parents cited HA dislodgement as the main challenge; in young children, this was primarily by hand owing to increasing motor skills, and in certain toddlers, this was due to temper tantrums.^10^ Furthermore, the degree of hearing loss and a higher level of family education were beneficial for prolonged HA use.^9^

Although hearing loss has recently received considerable attention in society, many families have reported that the use of HAs remains stigmatized.^11^ This study aimed to investigate the factors and conditions that could affect consistent HA usage in children.

## Methods

This study retrospectively analyzed the data of children with hearing impairment aged 6 months to 6 years who were fitted with HAs for at least 6 months. Data were collected from the audiology department. Patients with moderate to profound sensorineural hearing loss (SNHL) with complete data-logging information stored in the HA programming file from January 2020 until June 2021 were included. Children with concomitant disabilities were excluded. The study protocol was approved by the Institutional Review Board, Ref. N0.20/0021/IRB.

All audiological assessments, including hearing assessment through behavioral responses or auditory brain stem responses, aided sound field hearing thresholds at frequencies of 0.5, 1, 2, and 4 KHz, and aided speech detection threshold (SDT) or speech reception threshold (SRT), were retrieved from patient charts. The following demographic data were recorded: age/ gender of participant, parent factors (age, educational levels, and occupations of both parents), family factors (area of residence, family size, and birth order), factors related to family history of hearing loss (family history of hearing loss, HA use, or cochlear implantation), and lastly, factors related to the use of HAs (HA history, enrollment in school or daycare with HA, and duration of HA use).

To assess the consistency of HA usage at home, the HA programming software was checked for data-logging records at 3 and 6 months after the initial HA fitting. These records were compared with the parents’ reported average daily HA use at home. At the 6-month follow-up, parents were interviewed by the treating physician regarding the acceptance of, and satisfaction with, the HAs; signs of discomfort; battery consumption; esthetic appeal; and subjective performance at home. Parents were also asked whether the children requested the HAs. The following subgroups were formed: i) children who requested the HA themselves and those who did not, and ii) children who rejected the HA when placed on the ears. Missing data from the patient records were obtained by contacting the parents.

### Statistical analysis

Data analysis was carried out using Statistical Package for the Social Sciences, version 24 (IBM, Armonk, NY). Quantitative variables are presented as the mean ± standard deviation or the median (range). Qualitative variables are presented as frequencies and percentages. The Kolmogorov–Smirnov test was performed to determine the normality of the quantitative variables. Most variables were non-parametric. Non-parametric variables were compared between the 2 groups using the Mann–Whitney test and between more than 2 groups using the Kruskal–Wallis test, followed by the multiple Mann–Whitney test. Correlation among non-parametric variables was determined using Spearman’s rank correlation (rho). Statistical significance was set at *p*<0.05.

## Results

A total of 59 pediatric hearing aid users were included in this study. Table 1 summarizes the demographic characteristics of the participants. The average age was 40.8 ± 23.8 months (range: 10-120 months). All participants had SNHL, and 76% of the patients had severe to profound severity, 17% had moderately severe SNHL, 5% had moderate SNHL, and 2% had mild hearing loss. Twenty-one families reported a family history of hearing loss, and 17 of these had a history of cochlear implantation. The average age of the mothers was 33 ± 5.7 years, whereas that of the fathers was 38 ± 6.5 years. Approximately 60% of parents had at least a bachelor’s degree. Most fathers (93%) were employed, whereas most mothers (78%) were housewives. Approximately two-thirds of the families (68%) comprised at least 5 members, including both parents. Approximately half of the families (56%) resided in the Riyadh metropolitan area, whereas the remainder resided in various remote locations requiring lengthy trips.

**Table 1:** - Demographic data of the study participants (N=59).

Variables	Data
Age of participant, years, mean ± SD	40.83 ± 23.82
Age of father, years, mean ± SD	38.98 ± 6.56
Age of mother, years, mean ± SD	33.63 ± 5.71
Family size, n, mean ± SD	5.39 ± 2.17
Birth order, mean ± SD	3.08 ± 2.23
***Gender, %***	
Male	35 (59.3)
Female	24 (40.7)
***Residence, %***	
Riyadh	33 (55.9)
Outside Riyadh	26 (44.1)

SD: standard deviation

**Figure 1:**
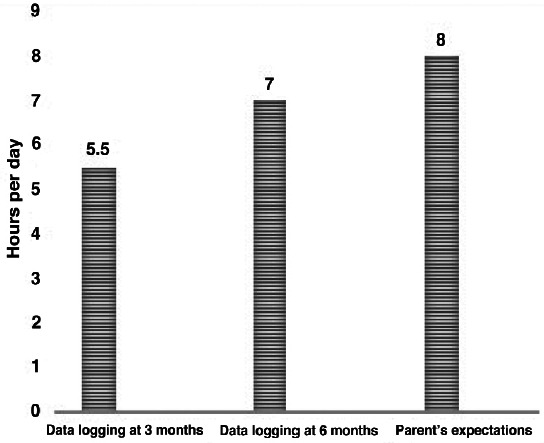
- Comparison of data logging at 3 and 6 months, and parental expectations of daily use.

Most children used bilateral HAs, and only 9 used unilateral HAs. The average period of HA use was 16.8 ± 15.8 months, and that reported by parents was 8.7 ± 4.7 hours (h)/day. Based on the log data, the average daily usage after 3 months was 5.5 h, and that after 6 months was 7 h (Figure 1). More than half of the children (56%) turned off their HAs ≤2 times per day. Females used HAs for longer periods than males; however, there was no significant difference in performance between the genders.

Approximately two-thirds (65%) of the families reported satisfaction with the esthetic appearance of HAs. From the information provided by the parents, signs of discomfort were present in 34% of the children, and 47% resisted using HAs. However, 46% of parents reported that their children requested to use their HAs. Children’s performance with HA use as reported by their parents, including their proportions, were as follows: excellent (34%), good (47%), and poor performance (19%). Audiological assessments revealed that the mean aided SDT for the right ear was 48.3 ± 18.7 dB HL and 48.2 ± 18.8 dB HL for the left ear, and the aided pure tone average (PTA) for the right ear was 48.8 ± 16.47 dB HL and 47.5 ± 17.27 dB HL for the left ear.

### Factors affecting daily use of HA

We investigated the factors that could affect the daily use of HAs and children’s performances using the Spearman rank correlation test (Table 2). Patient age was strongly positively correlated with both data-logging information and aided hearing threshold. The information obtained from the parents on how long their children used the HA each day was informative and correlated well with that obtained from data logging.

**Table 2:** - Correlation analysis of aided hearing threshold and data-logging records.

	Children’s age	Father’s age	Mother’s age	Family size	Duration of HA usage (months)	Parent’s estimation of daily use (hours)
Data log for 3 months	0.414**	0.123	0.192	0.060	0.325**	0.623*
Data log for 6 months	0.406**	0.064	0.205	0.014	0.416**	0.772**
Aided PTA	−0.416**	−0.146	−0.189	0.202	−0.451**	−0.312*
Aided SDT	−0.392**	−0.220	-0.202	0.157	−0.381**	−0.275*

Correlation coefficients are shown. HA: hearing aid, PTA: pure tone average for 4 frequencies (500, 1000, 2000, and 4000 Hz); SDT: speech detection threshold, **Correlation is significant at the 0.01 level (2-tailed). *Correlation is significant at the 0.05 level (2-tailed).

Regarding data-logging records, we found that a higher degree of education of the father (bachelor’s or higher) was a significant factor, whereas that of the mother was not. Notably, reduced HA usage was associated with lower education levels of parents.

Moreover, the higher the educational degree of the parents, the better the children’s performance. A moderate negative significant correlation between the parental rating for their children’s performance and the aided hearing threshold (r= -0.541, *p*=0.001) and a moderate positive correlation with the data-logging information (r=0.594, *p*=0.001) were noted.

**Figure 2:**
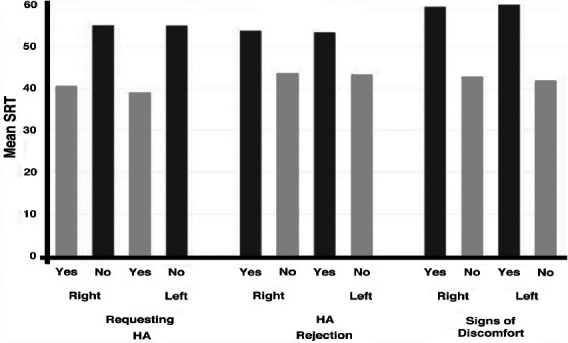
- Comparison of hearing thresholds (mean speech reception threshold [SRT]) in children who requested hearing aid (HA), who rejected HA, and who reported discomfort while wearing HAs.

**Figure 3:**
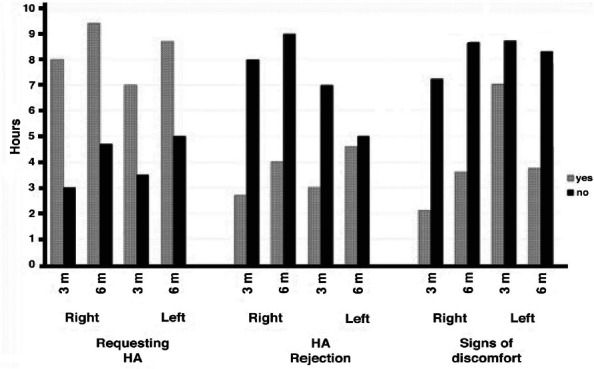
- Comparison of data logging information in children who requested hearing aid (HA), who rejected HA, and who reported discomfort while wearing HA according to the duration of use of HA (0–3 months, 3–6 months). m: months

Children who requested HAs possessed better data-logging information and higher hearing thresholds than the other patient groups (Figures 2 & 3). Similarly, there was a significant difference in the performance and the duration of daily usage (*p*=0.001) between the children who rejected the HA and those who accepted it. A comparison of the data-logging information showed that children who reported signs of discomfort had significantly longer hours (*p*=0.000) of daily HA use than those who did not. Moreover, children who consistently used their devices regardless of location and situation performed significantly better (*p*=0.000) in terms of daily HA use and hearing thresholds than those who only used them at home or at school.

### Subgroup analysis

The participants were divided into 3 subgroups according to age: <2 years, 2-4 years, and >4 years (Table 3). Significant differences between the 3 subgroups regarding the signs of discomfort and the rejection rate of the HA were noted, with higher values observed in younger children. A significant difference in data logging was only noted at 3 months of HA use; there was no significant difference at 6 months. Our results also showed that the performance of older children was significantly better than that of younger ones.

**Table 3:** - Comparison between the 3 subgroups regarding the aided hearing threshold, data logging, signs of discomfort, and hearing aid rejection.

Participant groups	Aided hearing threshold (mean ± SD)	Data log at 3 months (mean ± SD)	Data log at 6 months (mean ± SD)	Signs of discomfort		HA rejection		
				Yes	No	Yes	No	
Aged <2 years	66.6 ±15.4	3.2 ± 3.1	5.1 ± 4.4	11	5	14		2
Aged 2–4 years	55.5 ± 20.9	4.5 ± 4.1	5.9 ± 4.2	5	12	9		8
Aged >4 years	48.8 ± 16.1	7.6 ± 5.3	8.6 ± 5.2	4	22	5		21
*P*-value	0.02	0.039	0.083	<0.001*		<0.001*		

HA: hearing aid, SD: standard deviation. *Statistically significant differences.

## Discussion

This study primarily aimed to explore the factors that might affect the consistency of daily HA usage in the pediatric population, as reflected in the data-logging technology of modern HAs. We investigated 59 families of children with hearing loss who were using HAs. The families were from different backgrounds and socioeconomic levels. The first factor examined was the children’s age. Data-logging information improved with increasing age. This result is in agreement with the findings of Moeller et al,^10^ who revealed that the age of the child is a critical factor affecting HA usage. They reported that it was more difficult to maintain regular use of HAs in infants than in children aged >2 years. Another study reported that during infancy, maintaining consistent HA use is challenging and that preschool children use HAs for prolonged periods.^9^

We found that female children used HAs for longer periods than males. Similarly, Marnane and Ching^12^ reported less HA use in male children than in females, but with no apparent effect on performance. This might be due to the fact that girls are less likely to participate in activities, such as outdoor sports, that could pose a challenge to maintaining HAs.^12^ Additionally, the children residing in Riyadh had less data than those who lived outside Riyadh. We speculated that the parents of children residing in large cities, such as Riyadh, are usually employed and may have limited time to monitor their children during the day, whereas parents of those residing outside large cities could have more time for close monitoring.

Additionally, the father’s educational level had a greater influence on increasing HA use, and hence boosting children’s performance, than the mother’s. However, this is in contrast with a recent study reporting that the mother’s educational level is favorably linked with the duration of daily HA usage in children.^12^ We speculated that a higher paternal educational level is associated with a higher socioeconomic status and consequently more access to healthcare services. A strong correlation between lower socioeconomic levels and poor outcomes following hearing amplification has been reported.^13,14^ Notably, in our study, the percentage of employed mothers was only 23%, whereas that of employed fathers was 92%. This suggests that the father’s role in securing the family’s financial needs is greater than that of the mother and that housewives have more time than working mothers to assist their children in maintaining and monitoring HA use. In contrast to the findings of Walker et al,^9^ our data revealed that the age of the parents and the family size had no significant effect on HA use duration. Similarly, higher parental educational levels have been linked to children with hearing impairment attending mainstream schools after appropriate interventions for hearing impairment.^15^

In terms of hearing loss severity, Gustafson et al^16^ and Walker et al^9^ reported that the more severe the hearing loss, the longer the duration of HA use by children; however, in this study, the degree of hearing loss had no effect on the duration of HA use. This difference can be attributed to the fact that most of our participants had severe to profound SNHL. Other studies have reported that the parents of children with severe to profound hearing loss are eager to acquire HAs for their children quickly and continue to use them.^17,18^

In a study of children aged 6 months to 7 years, Tomblin et al^7^ revealed that participants who used HAs for a prolonged period had improved speech and language development, and had better-aided hearing threshold assessment. Our findings are consistent with this study, confirming that reduced HA use has a negative influence on the assisted PTA threshold and SDT.

Previous research has indicated that when parents try to predict the length of HA usage by their preschool children, they have unrealistic expectations,^9^ and that improved agreement between parents’ estimation and objective data was only observed in school-aged children.^16^ In this study, the parents’ estimate of HA use duration was in agreement with the data recorded by the device. We attribute this to the fact that most parents included in this study are well-educated and familiar with the benefits of HAs. Similarly, another study indicated that families with a higher socioeconomic or educational level have a greater degree of knowledge and awareness regarding hearing loss and its proper management.^15^

Participants used HAs at home or in school. When HAs were used in unmonitored places, participants were typically concerned about their hearing loss. The locations where children used their HAs on a frequent basis were studied by Walker et al^6^ Parents reported reduced HA use in infants under poor monitoring circumstances and increased HA use in older children in all locations, including in the car, daycare, and public places.^9^ We found that children who wore HAs at all times (namely, at home, outside, and in daycare or school) performed better than those who only wore them in one location.

This study revealed that signs of discomfort and rejection of Has strongly influence a shorter duration of HA use and subsequent poor performance. Parents’ complaints and concerns about the duration of HA use should be taken seriously. We found that the duration of HA use was not associated with esthetics, battery consumption, the number of HAs owned, or the number of HA maintenance visits per year.

The primary limitation of this study is the small sample size; thus, future studies should investigate a larger cohort with a longer duration of follow-up to validate our findings.

In conclusions, overall, we found that children’s age, gender, residential location, and parents’ educational level, particularly the father’s, had a significant effect on the duration of HA use and auditory performance. In contrast, family size, family history of hearing loss or the use of hearing amplification devices, and the parents’ ages or occupations had no discernible influence. Parental reports on the duration of HA use, the areas where they are used, the child’s acceptance of the HA, the child’s performance, and the symptoms of discomfort should all be considered. These findings emphasize the importance of parental input in HA use.
